# Right Ventricular–Protective Strategies in Pulmonary Embolism With Cardiopulmonary Failure: Prone Positioning and Mechanical Ventilation

**DOI:** 10.1016/j.jaccas.2026.110038

**Published:** 2026-08-28

**Authors:** Jorge Beauregard-Mora, Orlando Ruben Perez-Nieto, Carlos Mendiola-Villalobos, Jonathan Reyes-Montufar, Mateo Porres-Aguilar, Jose Antonio Meade-Aguilar, Tania Olga Mondragon-Labelle, Ernesto Deloya-Tomas

**Affiliations:** a Intensive Care Unit, Hospital General San Juan del Rio, Queretaro, Mexico; b Universidad Autonoma de Queretaro, Queretaro, Mexico; c Division of Vascular Medicine, Gonda Vascular Center, Mayo Clinic, Rochester, Minnesota, USA; d Division of Pulmonary and Critical Care Medicine, Mayo Clinic, Rochester, Minnesota, USA

**Keywords:** mechanical ventilation, prone position, pulmonary embolism, right ventricular failure, thrombolytic therapy

## Abstract

**Objective:**

We describe a reproducible bedside strategy to unload the right ventricle (RV) and improve oxygenation after systemic thrombolysis in intubated patients with pulmonary embolism (PE) and cardiopulmonary failure (category E1+R) in centers without extracorporeal membrane oxygenation.

**Key Steps:**

Confirm category E1+R PE and give weight-adjusted systemic thrombolysis. Use restrictive fluids and a diuretic to decongest the RV. Use vasopressors and minimal sedation to maintain arterial pressure and perfusion. Apply RV-protective ventilation: zero positive end-expiratory pressure, low tidal volume, controlled hyperventilation. Correct acidosis toward mild alkalemia with sodium bicarbonate. Add prone positioning as rescue for refractory hypoxemia.

**Potential Pitfalls:**

Potential pitfalls include post-thrombolysis bleeding, positive end-expiratory pressure–induced RV afterload, airway or line dislodgment during prone positioning, propofol-related hypotension, and overventilation despite embolic dead space.

**Take-Home Message:**

In category E1+R PE, this RV-protective strategy can support the patient through the perithrombolysis period of greatest severity in centers without extracorporeal membrane oxygenation.

## Visual Summary

**Figure:**
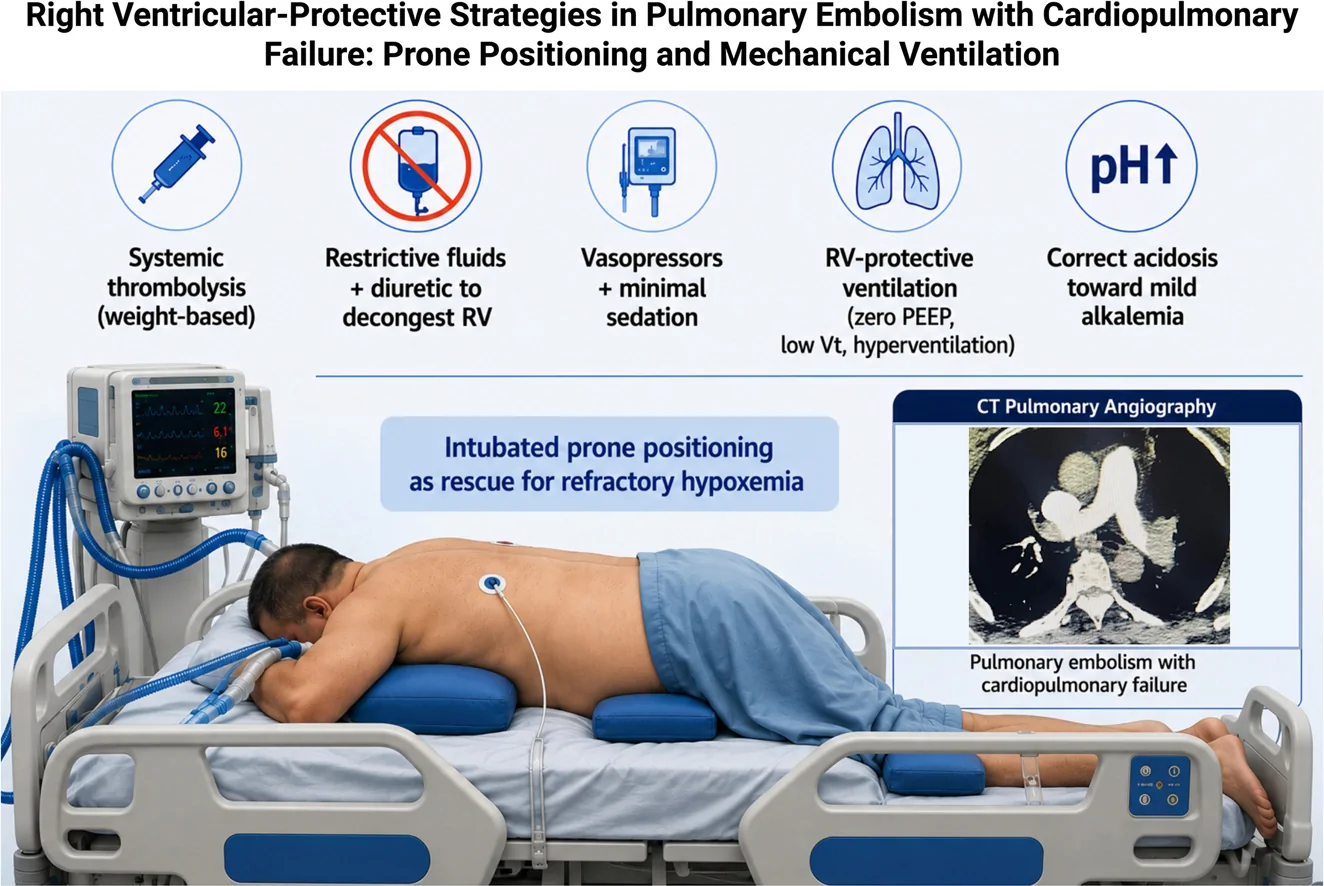

Under the 2026 American College of Cardiology/American Heart Association Acute Pulmonary Embolism Clinical Categories (A to E), category E1 denotes cardiopulmonary failure with cardiogenic shock, and the R modifier denotes hypoxemic or ventilatory failure.^1^ Category E1+R pulmonary embolism (PE) causes acute right ventricular (RV) pressure overload, reduced left ventricular filling, and obstructive shock, with high early mortality despite anticoagulation and reperfusion. In intubated patients, positive intrathoracic pressure, hypoxemia, and hypercapnia directly increase pulmonary vascular resistance (PVR) and RV afterload.^2^ Extracorporeal membrane oxygenation (ECMO) can stabilize the most severe cases, but it is unavailable in most centers worldwide. Here we describe a simple, reproducible, bedside strategy that any critical care team can apply after systemic thrombolysis: RV-protective ventilation, acid-base optimization toward mild alkalemia, and prone positioning as rescue for refractory hypoxemia. We illustrate the approach with 2 consecutive patients in whom PE was managed with an RV-centered protective strategy.

## Case Summary

Patient 1 was a 64-year-old man in whom PE progressed within hours from category C2+R (abnormal RV without abnormal biomarkers, plus the respiratory modifier) to category E1+R, with refractory hypoxemia and cardiogenic shock. After noninvasive ventilation failed, he was intubated with an RV-protective strategy (zero positive end-expiratory pressure [PEEP], controlled hyperventilation). He received weight-adjusted tenecteplase (45 mg), norepinephrine and vasopressin, restrictive fluids, and intravenous sodium bicarbonate (89 mEq) to target mild alkalemia (pH 7.52). Prone positioning was used as rescue for life-threatening hypoxemia, with rapid improvement in oxygenation and hemodynamics and no bleeding. He was weaned, extubated to high-flow nasal cannula, and discharged on rivaroxaban (Figures 1A to 1D).

**Figure 1:**
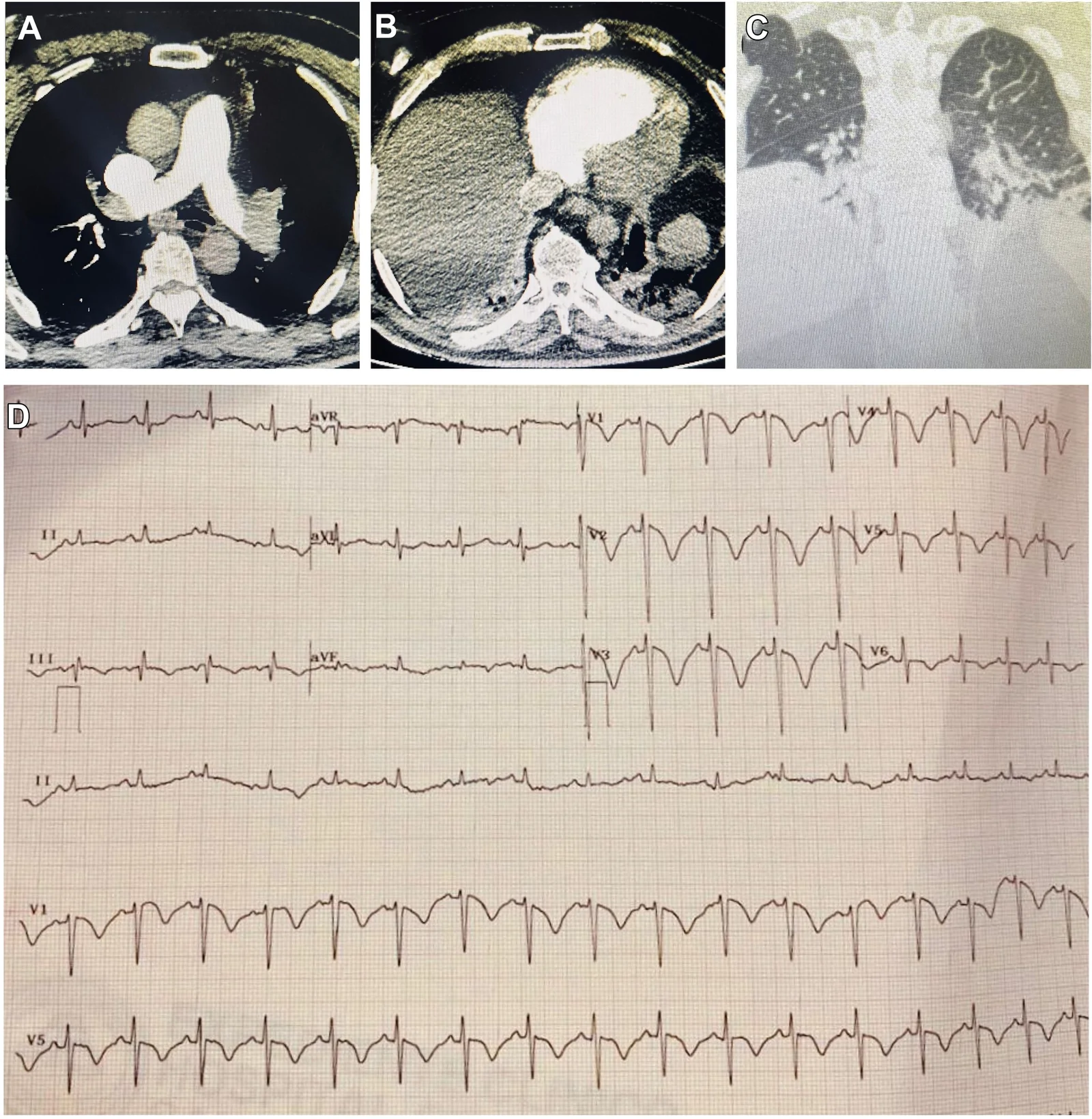
Patient 1 (A) Computed tomography pulmonary angiography showing acute bilateral central pulmonary thromboembolism with bilateral intraluminal filling defects involving the main pulmonary artery branches at the pulmonary trunk bifurcation. (B) Axial cardiac-level computed tomography pulmonary angiography showing right-sided chamber dilation, particularly of the right ventricle, with interventricular septal flattening and mild leftward bowing, consistent with acute right ventricular overload and ventricular interdependence. (C) Coronal chest computed tomography (lung window) showing a wedge-shaped, pleural-based opacity in the left lower lobe consistent with pulmonary infarction. (D) 12-lead electrocardiogram (paper speed 25 mm/s; gain 10 mm/mV) showing sinus tachycardia (approximately 120-130 beats/min) and an S1Q3T3 pattern, consistent with acute right ventricular strain.

Patient 2 was a 36-year-old man with obesity admitted with category C3+R PE (abnormal RV and an elevated natriuretic peptide, plus the respiratory modifier), comprising peripheral (segmental and subsegmental) emboli, left upper-limb deep vein thrombosis, and left jugular vein thrombosis, with severe RV dysfunction (left ventricular ejection fraction 22%-24%); over the following days he progressed to category E1+R. He was intubated with zero PEEP RV-protective ventilation; despite controlled hyperventilation, Paco_2_ reached 63 mm Hg, reflecting embolic dead space. He received alteplase (100 mg over 2 hours), dual vasopressors, and sodium bicarbonate (89 mEq). For refractory hypoxemia, the day after thrombolysis, a single prone session at zero PEEP improved the Pao_2_/Fio_2_ ratio from 61 mm Hg to 93 mm Hg and converted acidemia to mild alkalemia (pH 7.31-7.47) (Figures 2A to 2D). He recovered and was discharged on apixaban.

**Figure 2:**
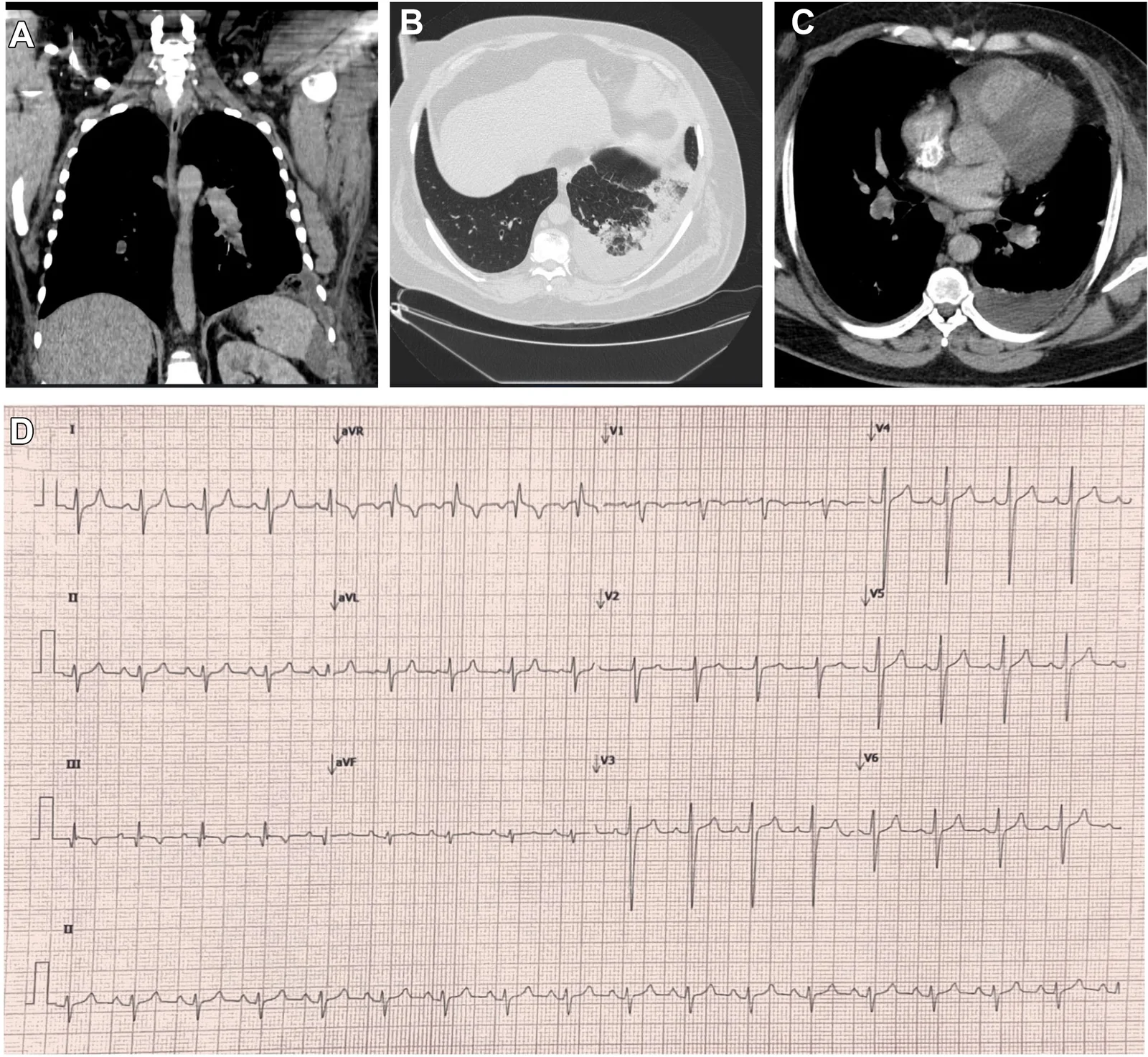
Patient 2 (A) Coronal contrast-enhanced computed tomography reconstruction showing dilation of the central pulmonary arteries and a left basal, pleural-based parenchymal opacity. (B) Axial computed tomography of the lower thorax (lung window) showing dense consolidation with surrounding ground-glass opacity in the left lower lobe, adjacent to the diaphragm and hepatic dome, consistent with pulmonary infarction with an associated inflammatory or pneumonic process, together with a small left pleural effusion. (C) Axial contrast-enhanced computed tomography at the level of the pulmonary trunk and cardiac chambers showing segmental and subsegmental intraluminal filling defects with dilation of the main pulmonary artery, right ventricular dilation, and septal flattening, consistent with acute right ventricular overload and ventricular interdependence. (D) 12-lead electrocardiogram (paper speed 25 mm/s; gain 10 mm/mV) showing sinus tachycardia (approximately 100-110 beats/min), an S1Q3T3 pattern, and T-wave inversions in the right precordial leads (V_1_ to V_3_), consistent with acute right ventricular strain.

## Procedural Steps

### Confirm PE With Cardiopulmonary Failure

Use bedside point-of-care ultrasound (RV dilation, septal flattening, McConnell sign, reduced tricuspid annular plane systolic excursion) and, when feasible, computed tomography pulmonary angiography. Support the diagnosis with an elevated D-dimer, RV strain on the electrocardiogram (S1Q3T3, right precordial T-wave inversions), and validated risk scores. Confirm hypotension or shock to establish cardiopulmonary failure, and classify severity with the clinical categories; both patients reached category E1+R, compatible with Society for Cardiovascular Angiography and Interventions SHOCK stage C.^1^

### Deliver Systemic Thrombolysis Promptly

Give a weight-adjusted single bolus of tenecteplase or alteplase 100 mg infused over 2 hours, after checking for absolute contraindications and obtaining consent.^3^ Minimize venipunctures and invasive procedures around administration to reduce bleeding.

### Decongest RV: Restrictive Fluids and Diuresis

Use a restrictive fluid strategy to avoid RV overdistention. When there is RV volume overload or venous congestion and blood pressure allows, add a loop diuretic to decongest and offload the RV; avoid diuresis in preload-dependent hypotension.

### Support Hemodynamics

Start norepinephrine, adding vasopressin if needed, to keep mean arterial pressure at least 65 mm Hg and preserve RV coronary perfusion. Keep sedation to a minimum to limit vasodilation and myocardial depression.

### Intubate Only When Necessary, Using an RV-Protective Approach

Preoxygenate with noninvasive ventilation, and elevate the head of the bed. Avoid propofol; use fentanyl with midazolam or etomidate, plus a rapid-onset neuromuscular blocker. Anticipate hemodynamic collapse and have vasopressors ready.

### Set RV-Protective Ventilation

Use zero PEEP during the most unstable phase, low tidal volume (about 6-8 mL/kg predicted body weight), high Fio_2_, and controlled hyperventilation (minute ventilation about 200 mL/kg/min, roughly 14 L/min).^4^ Expect Paco_2_ to remain elevated despite high minute ventilation because of embolic dead space; do not aggressively maintain normocapnia by increasing mechanical ventilator settings to injurious pressures.^5^

### Optimize Acid-Base Status Toward Mild Alkalemia

Correct hypoxemia, hypercapnia, and acidosis, all of which raise PVR. Administer intravenous sodium bicarbonate (eg, 10 ampules of 7.5%, about 89 mEq) to reach a pH near 7.45 to 7.50, attenuating hypoxic pulmonary vasoconstriction and unloading the RV.

### Apply Prone Positioning as Rescue for Refractory Hypoxemia

When oxygenation remains life threatening following the above-mentioned measures and ECMO is unavailable, turn the patient prone while maintaining zero PEEP.^6^ Obtain arterial blood gas test immediately before prone positioning and again at 12 to 24 hours. Secure the endotracheal tube, central and dialysis lines, and pressure points before turning.^7^^,^^8^

### Monitor, wean, and De-escalate

Track the arterial blood gases, mean arterial pressure, vasopressor dose, and lactate. As the RV recovers and oxygenation improves, return the patient to supine position, reduce sedation, and follow a standardized weaning protocol. Extubate to high-flow nasal cannula, adding nocturnal noninvasive ventilation when obstructive sleep apnea is present (Table 1).

**Table 1:** Equipment and Recommended Settings for Right Ventricle–Protective Strategy in Pulmonary Embolism With Cardiopulmonary Failure (Category E1+R)

Item	Setting or Specification
Thrombolytic	Tenecteplase weight-based single bolus, or alteplase 100 mg over 2 h
Ventilator	Zero PEEP; tidal volume 6-8 mL/kg predicted body weight; high Fio_2_; controlled hyperventilation (minute ventilation approximately 200 mL/kg/min)
Fluids and diuresis	Restrictive fluid strategy; loop diuretic when RV volume overload or venous congestion and blood pressure allows
Vasopressors	Norepinephrine, plus vasopressin if needed; target mean arterial pressure at least 65 mm Hg
Induction and sedation	Fentanyl with midazolam or etomidate; rocuronium; avoid propofol
Acid-base	Sodium bicarbonate 7.5%; target pH 7.45-7.50
Monitoring	Serial blood gases; point-of-care ultrasound or echocardiography; lactate
Prone positioning	Trained team of at least 5; endotracheal tube and femoral line securement; pressure-point padding
Oxygen backup	High-flow nasal cannula; CPAP or noninvasive ventilation for weaning and for obstructive sleep apnea

Thrombolytic choice (tenecteplase or alteplase) depends on local availability.

CPAP = continuous positive airway pressure; PEEP = positive end-expiratory pressure; RV = right ventricular.

## Potential Pitfalls

### Bleeding After Thrombolysis

Avoid nonessential punctures and catheter placements around thrombolysis, monitor neurologically, and hold any anticoagulant bolus until bleeding risk is reassessed.

### PEEP-Induced RV Afterload

PEEP can raise intrathoracic pressure and PVR; default to low or zero PEEP during instability and titrate cautiously only after recovery.

### Pursuing Normocapnia

Aggressive hyperventilation raises airway pressure and can worsen RV afterload. Because dead space keeps Paco_2_ high, accept permissive hypercapnia rather than escalate pressures.

### Sedation Choice

Propofol causes vasodilation and myocardial depression and has been linked to worse outcomes during PE interventions. Fentanyl with midazolam or etomidate is preferred.^9^

### Prone Positioning Complications

Coordinate a trained team, secure the airway and all lines (including femoral dialysis catheters), protect pressure points, and screen for contraindications such as spinal instability or an open abdomen.

### Volume Overload

Liberal administration of fluids distends the failing RV; use a restrictive strategy and vasopressors instead.

### Bicarbonate Cautions

Rapid alkalinization can lower ionized calcium and potassium and generate carbon dioxide. Monitor electrolytes and ensure adequate ventilation to clear the carbon dioxide load.

## Conclusions

In centers without ECMO, a physiologically guided, RV-protective strategy can bridge intubated patients with PE with cardiopulmonary failure through the critical perithrombolysis window. The core elements are simple and reproducible: zero PEEP RV-protective ventilation, correction of hypoxemia and acidosis toward mild alkalemia, restrictive fluids and diuresis with vasopressor support, and prone positioning as rescue for refractory hypoxemia. In 2 consecutive patients, this approach improved oxygenation and hemodynamics without bleeding complications. Prospective study is warranted, ideally within multidisciplinary pulmonary embolism response teams.^10^ Both patients reached clinical category E1+R, and, consistent with the guideline preference for direct oral anticoagulants, each was transitioned to a direct oral anticoagulant for maintenance therapy (Table 2).^1^

**Table 2:** Deidentified Combined Timeline of Both Patients Illustrating the Right Ventricle–Protective Strategy After Systemic Thrombolysis in Pulmonary Embolism With Cardiopulmonary Failure (Category E1+R)

Timeline	Events
Patient 1 (64-y-old man; systemic thrombolysis with tenecteplase)	
Day 1	A 64-y-old man with probable chronic heart failure and remote severe traumatic brain injury presented with sudden severe dyspnea, oppressive chest pain, and profound hypoxemia (oxygen saturation near 65% on room air). Point-of-care ultrasound showed RV dilation, ventricular interdependence, and McConnell sign; echocardiography revealed a mobile thrombus in transit in the right side of the heart; CT pulmonary angiography confirmed bilateral central pulmonary embolism (category C2+R). Enoxaparin was started.
Day 1 (h later)	Noninvasive ventilation was attempted but failed, prompting rapid-sequence intubation with zero PEEP RV-protective ventilation and controlled hyperventilation. He progressed to cardiopulmonary failure (category E1+R) with severe hypoxemia (oxygen saturation 66%), hypotension (mean arterial pressure 64 mm Hg), mottling, and metabolic acidosis. Norepinephrine and vasopressin were started, with intravenous sodium bicarbonate (89 mEq) to target mild alkalemia (pH 7.52).
Day 1 (continued)	Weight-adjusted tenecteplase (45 mg) was administered, and prone positioning was used as rescue for life-threatening hypoxemia, with rapid improvement in oxygenation and hemodynamics and no bleeding.
Subsequent days	After a successful spontaneous breathing trial, he was extubated to high-flow nasal cannula and then conventional nasal cannula, with early mobilization.
Discharge	He was discharged on rivaroxaban, with cardiology, pulmonology, and primary care follow-up.
Patient 2 (36-y-old man; systemic thrombolysis with alteplase)	
Day 1	A 36-y-old man with obesity, hypertension, and obstructive sleep apnea presented with sudden severe chest pain and hypoxemia (oxygen saturation nadir near 70%). Electrocardiogram showed no ST-segment elevation, with negative troponin and positive myoglobin. Echocardiography and CT pulmonary angiography confirmed acute bilateral pulmonary embolism. He was discharged on a direct oral anticoagulant.
Day 3	He returned within 48 h after referral with hypoxemic failure (oxygen saturation 81%), tachycardia, and hypertension (category C3+R). Therapeutic enoxaparin was started.
Day 4	Imaging confirmed bilateral pulmonary thromboembolism with main pulmonary artery dilation, splenic infarction, an incidental solitary kidney, and left upper extremity deep vein thrombosis. Echocardiography showed severe RV dysfunction with a left ventricular ejection fraction of 22%-24%, consistent with acute cor pulmonale.
Day 5	He was admitted to the intensive care unit for close monitoring.
Day 7	Progressive respiratory distress and hemodynamic deterioration marked progression to category E1+R, prompting intubation with zero-PEEP RV-protective ventilation and controlled hyperventilation; dual vasopressors were started.
Day 8	Systemic thrombolysis with alteplase (100 mg over 2 h) was administered under close bleeding surveillance, with no major bleeding.
Day 9	For persistent life-threatening hypoxemia, a single prone session at zero PEEP was performed, with sodium bicarbonate (about 89 mEq) to target mild alkalemia. Over 24 h, Pao_2_/Fio_2_ ratio improved from 61 mm Hg to 93 mm Hg, and pH rose from 7.31 to 7.47.
Day 19	After 12 d of invasive mechanical ventilation, he was weaned and extubated to high-flow nasal cannula, then to nocturnal noninvasive ventilation for obstructive sleep apnea.
Day 26	He was transferred to the ward neurologically intact, with recovered renal function and no vasopressor requirement, after a 22-d intensive care stay and was discharged on apixaban.

Generic, unidentifiable timeline descriptors are used to avoid patient identification. Day numbers are relative to each patient's initial presentation rather than calendar dates.

CT = computed tomography; PEEP = positive end-expiratory pressure; RV = right ventricle/ventricular.

**Visual Summary:**
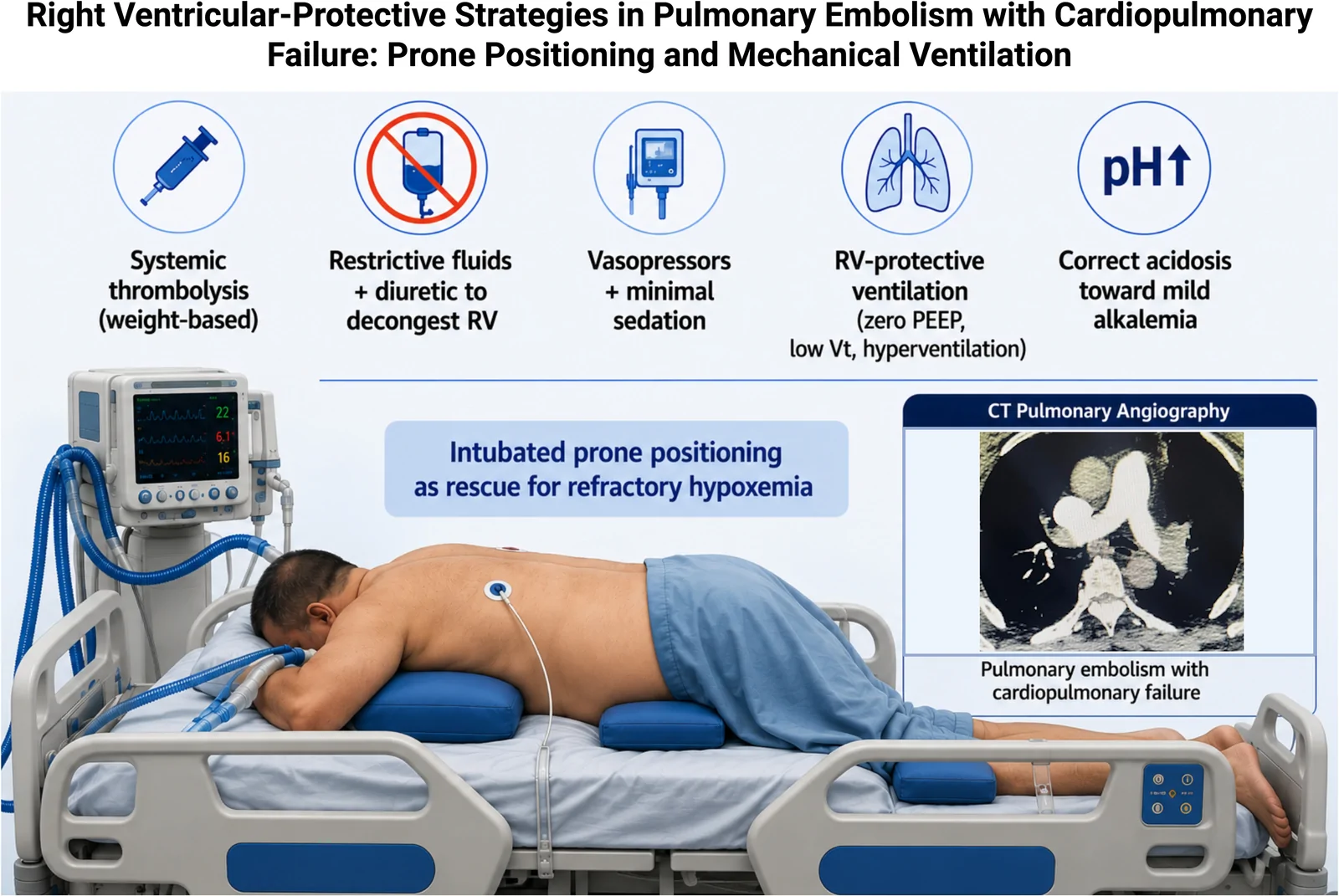
RV-Protective Bedside Strategy for Critically Ill Patients With Acute Pulmonary Embolism (Category E1+R) This figure summarizes a physiology-driven bedside strategy applied after confirmation of category E1+R pulmonary embolism (cardiopulmonary failure with the respiratory modifier) in centers without extracorporeal membrane oxygenation. (Top row, from left to right) Weight-based systemic thrombolysis to restore pulmonary blood flow; restrictive fluids with a loop diuretic to decongest the right ventricle (RV); vasopressors with minimal sedation to preserve arterial pressure and RV coronary perfusion; RV-protective ventilation with zero positive end-expiratory pressure (PEEP), low tidal volume (Vt), and controlled hyperventilation; and correction of acidosis toward mild alkalemia. (Center) Intubated patient in prone positioning as rescue for refractory hypoxemia, maintained at zero PEEP, improving ventilation-perfusion matching and reducing RV afterload. (Inset) Axial computed tomography (CT) pulmonary angiography showing an intraluminal filling defect consistent with acute pulmonary embolism. Collectively, these interventions target the reversible determinants of pulmonary vascular resistance, namely, hypoxemia, hypercapnia, acidosis, elevated intrathoracic pressure, and volume overload. Because pulmonary embolism produces substantial alveolar dead space, hypercapnia may persist despite adequate ventilation; normalization of Paco_2_ should not be pursued at the expense of RV protection. Figure created with BioRender.com; the central illustration was generated using the image engine of ChatGPT (OpenAI) and integrated and labeled in BioRender.

### Data Statement

The visual summary illustration was created using BioRender.com and OpenAI ChatGPT (GPT-5.5) image generation. The authors reviewed, edited, and approved the final content and assume full responsibility for its scientific accuracy and interpretation.

**Take-Home Message**

• In patients with clinical category E1+R PE, an RV-protective strategy combining systemic thrombolysis, zero PEEP, controlled hyperventilation, prone positioning, alkalinization with sodium bicarbonate, and diuresis can support the patient through the perithrombolysis period of greatest severity in centers without ECMO.

## Funding Support and Author Disclosures

The authors have reported that they have no relationships relevant to the contents of this paper to disclose.
